# Autophagy Participates in Lysosomal Vacuolation-Mediated Cell Death in RGNNV-Infected Cells

**DOI:** 10.3389/fmicb.2020.00790

**Published:** 2020-04-30

**Authors:** Youhua Huang, Ya Zhang, Zetian Liu, Chuanhe Liu, Jiaying Zheng, Qiwei Qin, Xiaohong Huang

**Affiliations:** ^1^College of Marine Sciences, South China Agricultural University, Guangzhou, China; ^2^Guangdong Laboratory for Lingnan Modern Agriculture, Guangzhou, China; ^3^Instrumental Analysis & Research Center, South China Agricultural University, Guangzhou, China; ^4^Laboratory for Marine Biology and Biotechnology, Qingdao National Laboratory for Marine Science and Technology, Qingdao, China

**Keywords:** RGNNV, vacuolization, lysosome, autophagy, cell death

## Abstract

Nervous necrosis virus (NNV) is the etiological agent of viral nervous necrosis (VNN), also known as viral encephalopathy and retinopathy (VER), which results in heavy economic losses to the aquaculture industry worldwide. Dramatic cytoplasmic vacuoles were observed during NNV infection both *in vitro* and *in vivo*; however, the origin and mechanism of cytoplasmic vacuolization remains unknown. In this report, we found that the cytoplasmic vacuole morphology became fused and enlarged during infection with red spotted grouper nervous necrosis virus (RGNNV), which was accompanied by increased cell death. Notably, Lyso-Tracker, but not Mito-Tracker or ER-Tracker, was accumulated in the vacuoles, and abnormal lysosome swelling was observed in RGNNV-infected cells, suggesting that the cytoplasmic vacuoles originated from lysosomal organelles. Cytoplasmic vacuolization and cell death in RGNNV-infected cells was completely blocked by the vacuolar H^+^-ATPase inhibitor (bafilomycin A1), and was significantly weakened by chloroquine (CQ), a lysosomotropic agent that induces the acidification of the lysosomes. This suggests that lysosome acidification was essential for vacuole formation. Significant inhibitory effects on vacuolization and cell death were also observed in the RGNNV-infected cells following treatment with nigericin and monensin (ionophores that uncouple the proton gradient present in lysosomes). This indicated that lysosome function was tightly associated with RGNNV infection-induced cell death. In addition, vacuoles were found to be partially co-localized with GFP-LC3II punctate dots during RGNNV infection. Moreover, the severity of vacuolization and cell death were both significantly decreased after treatment with the autophagy inhibitor, 3-MA, suggesting that autophagy was involved in lysosomal vacuolization and cell death evoked by RGNNV infection. Thus, our results demonstrate that autophagy participates in lysosomal vacuolation-mediated cell death during RGNNV infection, and provides new insight into our understanding of the potential mechanisms underlying nodavirus pathogenesis *in vitro*.

## Introduction

Viral nervous necrosis (VNN), otherwise termed viral encephalopathy and retinopathy (VER), caused by nervous necrosis virus (NNV) (genus *Betanodavirus*, family *Nodaviridae*) is a highly infective neuropathological disease that can be detected in more than 177 marine species worldwide (Costa and Thompson, 2016; Doan et al., 2017; Bandín and Souto, 2020). Moreover, NNV infection causes more than 90% mortality in several marine cultured fish species at the larval and juvenile stages (Parameswaran et al., 2007). Currently, betanodaviruses are classified into four genotypes based on the RNA2 sequence: (1) red-spotted grouper NNV (RGNNV); (2) barfin flounder NNV (BFNNV); (3) tiger puffer NNV (TPNNV); and (4) striped jack NNV (SJNNV) (Nishizawa et al., 1997) with a proposed fifth, turbot NNV (TNNV) (Johansen et al., 2004), and three other known unclassified viruses (Sahul Hameed et al., 2019). Strains belonging to the RGNNV genotype cause a high mortality in the grouper industry in many countries (Hegde et al., 2002), and evoke mass cytoplasmic vacuolization in the retina and brain of infected fish (Chi et al., 1997). Moreover, the numerous cytoplasmic vacuoles are also observed in RGNNV-infected cells (Huang et al., 2011); however, the origin and potential mechanism of vacuolization during NNV infection remains poorly understood.

Cytoplasmic vacuolization, commonly termed vacuolation, is an acknowledged morphological phenomenon observed in mammalian cells both *in vivo* and *in vitro* during exposure to bacterial or viral pathogens, as well as to various drugs and other substances (Aki et al., 2012; Shubin et al., 2016). To date, the vacuolization effects caused by viral infection have been investigated in members of 15 viral families, including hepatitis A virus (HAV), hepatitis C virus (HCV), bovine virus diarrhea virus (BVDV), murine leukemia virus (MuLV), Zika virus, hepatitis B virus (HBV), and polyomaviruses (Shubin et al., 2016; Monel et al., 2017). Viral products (e.g., enveloped or capsid proteins) have been shown to act as vacuolization inducers (Shubin et al., 2015; Luo et al., 2016), and the mechanisms underlying the vacuolization effects differ. For example, 3C protease of hepatitis A virus (3Cpro) has induced numerous non-acidic cytoplasmic vacuoles, which were originated from the endosome and lysosome compartments (Shubin et al., 2015). Moreover, simian virus 40 (SV40) induces substantial cytoplasmic vacuoles at the late productive infection stage, and the binding of viral major capsid protein VP1 to the cell surface ganglioside, GM1, triggers the formation of cytoplasmic vacuoles (Murata et al., 2008; Luo et al., 2016).

Vacuolization evoked by an exogenous stimulus has been demonstrated to be derived from different membrane organelles, including mitochondria, endoplasmic reticulum (ER), lysosome, Golgi apparatus, and autolysosomes (Aki et al., 2012). Moreover, vacuolization usually accompanies different types of cell death, such as paraptosis-like cell death, necroptosis, and autophagy-associated cell death (Shubin et al., 2015; Monel et al., 2017). Therefore, an investigation of the vacuole origin and properties will contribute to elucidating the mechanisms of the pathomorphological effects of vacuolization inducers. For example, the MuLV envelope protein (Env)-induced cytoplasmic vacuoles were derived from the ER, and partially formed from fused endosomal/lysosomal organelles and autophagosomes (Whatley et al., 2008). During HBV infection, the large HBV surface antigen (L-HBsAg) was also found to trigger ER vacuolization (Foo et al., 2002), whereas the vacuolating effect of L-HBsAg appears to be the cause of cell death (Xu et al., 1997). In addition, BVDV infection induces vacuolization of acidic endosomal/lysosomal organelles, and the formation of vacuoles and cell death is autophagy-independent (Birk et al., 2008).

In the present study, we investigated the origin of the vacuoles triggered by an infection with RGNNV in grouper cells. Furthermore, the critical factors and events involved in vacuole formation and cell death were clarified. Together, our data will both shed important light on the characteristics of RGNNV-induced vacuolization and cell death, as well as contribute to our understanding of the mechanisms of nodavirus pathogenesis.

## Materials and Methods

### Cell Culture, Virus, and Reagents

Grouper spleen (GS) cells were established and maintained in our lab (Huang et al., 2009). GS cells were grown in Leibovitz’s L15 medium containing 10% fetal bovine serum (Gibco) at 28°C. The RGNNV used in the study was prepared as described previously (Huang et al., 2011). For RGNNV infection, the GS cells were infected with RGNNV at a multiplicity of infection (MOI) of 2.

Monensin sodium salt (an ionophore that mediates Na+/H+ exchange) and nigericin sodium salt (a K+/H+ ionophore) were purchased from MedChemExpress (MCE). z-FA-FMK (inhibitor of cysteine proteases, including cathepsins B, S, and L) was purchased from Selleck. Chloroquine (CQ), bafilomycin A1 (Baf), E64D (L-trans-epoxysuccinyl (OEt)-leu-3-methylbutylamide-ethyl ester, pan-cysteine cathepsin inhibitor), and CA-074 (L-trans-epoxysuccinyl-Ile-Pro-OH propylamide, an inhibitor of cathepsin B) were purchased from Sigma-Aldrich. All reagents were dissolved in DMSO. 3-Methyladenine (3-MA) was purchased from Selleck and dissolved in sterile water. Lyso-Tracker (Red DND-99), Image-it dead green viability stain, Mito-Tracker (Red CMXRos), and ER-Tracker (Red) were obtained from Invitrogen. In addition, the plasmids, pEGFP-N3 (control vector), pEGFP-LC3 (GFP-tagged LC3 plasmid, a versatile marker of autophagy), pEGFP-Rab5 (marker for the early endosome), and pEGFP-Rab7 (marker for the late endosome), used in this study were stored in our lab as previously described (Wang et al., 2014).

### Virus Infection

GS cells were grown in either 24- or 6-well plates pretreated with DMSO, water, or different reagents (the optimal concentration used in this study was determined using a cell viability assay) for 2 h. The GS cells were infected with RGNNV at a MOI of 2 and cultured at 28°C. At 24 h post-infection (p.i.), the cytopathic effect (CPE) of the cells was observed under microscopy (Zeiss).

### Cell Viability Assay

To evaluate cell viability, cells treated with DMSO- or different reagents (Z-FA-FMK, CA-074, Baf, CQ, Monensin, Nigericin or 3-MA) were incubated with Image-It Dead green viability stain for 15 min, and the cells were imaged under a fluorescence microscope.

The percentage of cell death was also determined by trypan blue exclusion (Mullick et al., 2013). Briefly, the cells were collected by trypsinization and stained with trypan blue. Cell mortality (%) was presented as the percentage of dead cells out of the total number of cells.

### Evaluation of Autophagy

The effects of 3-MA on RGNNV-induced autophagy was determined using a Cyto-ID Autophagy detection kit (Enzo life sciences) as described previously (Huang et al., 2015). Briefly, the cells were seeded into 24-well plates at ∼80% confluence. Following treatment with 2 or 5 mM 3-MA, the cells were infected with RGNNV for 24 h, washed once in fresh medium, and subsequently stained with Cyto-ID green detection reagent for 30 min. Finally, the expression of bright green fluorescence in the vesicles was observed under a fluorescence microscope (Zeiss).

### Electron Microscopy

Mock- and RGNNV-infected cells were harvested at 24 h p.i. and 48 h p.i., and were washed with PBS. The cell pellets were fixed in 2.5% glutaraldehyde overnight. Sample preparation was performed as previously described (Huang et al., 2009). Briefly, after washing with PBS, the cells were post-fixed in 1% osmium tetroxide (OsO4) for 1 h, and then dehydrated in graded ethanol. Then cells were embedded in Epon resin. Sections were double stained with uranyl acetate and lead citrate. The grids containing ultrathin sections were examined using a Talos L120C electron microscope (Thermo Fisher Scientific) at 120 KV and micrographics were obtained using a CDD camera.

### Cell Transfection

Transfection was performed using Lipofectamine 2000 (Invitrogen) according to the manufacturer’s instruction as previously described (Huang et al., 2009). Briefly, GS cells were seeded into 24-well plates for 18 h, after which the cells were transfected with a mixture of Lipofectamine 2000 and pEGFP-N3, pEGFP-LC3, pEGFP-Rab7, or pEGFP-Rab5, respectively. After 24 h from the time of transfection, cells were infected with RGNNV for another 24 h, and then fixed in 4% paraformaldehyde for 1 h at 4°C. Finally, cells were stained with 1 μg/mL of 6-diamidino-2-phenyl-indole (DAPI, Sigma), and then observed under fluorescence microscopy (Zeiss).

### Immunofluorescence Assay

To evaluate protein synthesis during RGNNV infection, CP protein expression was detected using an immunofluorescence assay as described previously (Zheng et al., 2019). In brief, GS cells were grown in a 24-well plate overnight. The cells were pretreated with various reagents (CQ, Baf, Monensin or Nigericin), and infected with RGNNV in the presence of these reagents for an additional 24 h. Both mock- and RGNNV-infected treated cells were fixed in 4% paraformaldehyde at room temperature for 1 h, and then permeabilized with 0.2% triton X-100 for 15 min. After blocking with 2% bovine serum albumin (BSA), cells were incubated with rabbit anti-CP serum (1:300) (prepared in our lab) for 2 h, followed by the second antibody anti-rabbit IgG Fab2 Alexa Fluor 488 (1:200; Molecular probe). Finally, the cells were stained with DAPI and observed under a fluorescence microscope.

### RNA Extraction, cDNA Synthesis, and Quantitative PCR (qPCR)

To determine the effects of different reagents on RGNNV replication, the transcript of CP (fragment from 391–621 nn) was detected by qPCR. In brief, mock- or RGNNV-infected cells were collected, and the total RNA was extracted using an SV total RNA isolation system (Promega) according to the manufacturer’s instructions. The RNA was reverse transcribed using a ReverTra Ace qPCR RT Kit (TOYOBO). Amplification was examined using a SYBR Green I Reaction Mix (Toyobo) in an Applied Biosystems QuantStudio 5 Real Time Detection System (Thermofisher, United States). Each assay was carried out under the following cycling conditions: 95°C for 1 min for activation, followed by 40 cycles at 95°C for 15 s, 60°C, for 15 s, and 72°C for 45 s. The primers used in the experiment were those that were described previously (Zhang et al., 2019). The level of target gene expression normalized to β-actin was calculated using the 2^–ΔΔCT^ method. The data are representative of one representative experiment carried out in triplicate.

### Statistical Analysis

The results are presented as the mean ± standard deviation (SD). Statistical comparisons were performed using a Student’s *t*-test, and the statistical differences between groups were considered to be significant (^∗^) if the *p*-value < 0.05.

## Results

### Cytoplasmic Vacuolation Is a Typical Cytopathic Effect Induced by RGNNV Infection

**FIGURE 1:**
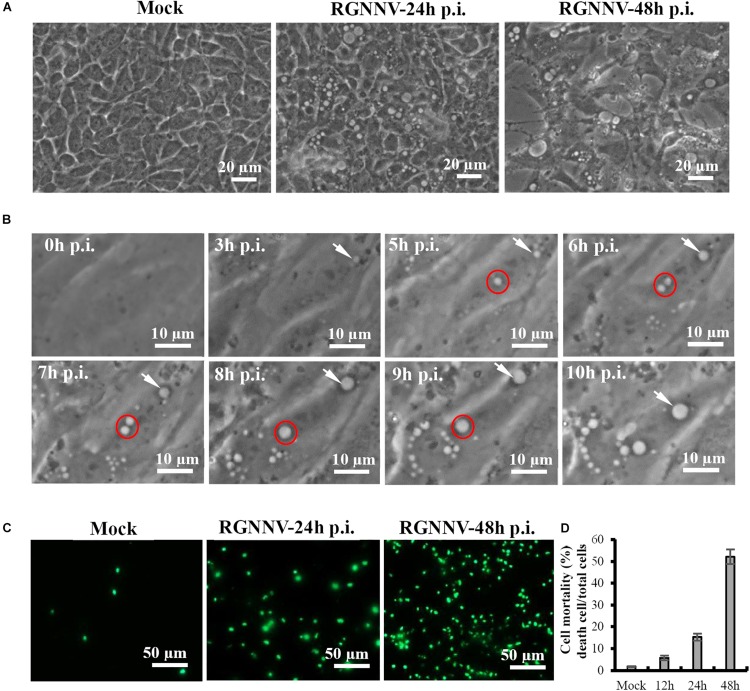
RGNNV induced massive cytoplasmic vacuoles in grouper cells. **(A)** RGNNV induced massive cytoplasmic vacuoles in GS cells. **(B)** Time-course observation of vacuoles from 3 to 10 h during RGNNV infection. The arrows indicate enlargement of vacuoles. The circles show the fusion of the vacuoles. **(C)** Cell death induced by RGNNV infection was determined by Image-It Dead green viability staining. **(D)** The percentage of the dead cells were quantified using a trypan blue assay.

We first performed a detailed investigation of the characteristics of vacuolization evoked by RGNNV infection. As shown in Figure 1A, a large number of vacuoles were observed in RGNNV-infected GS cells at 24 h p.i. At 48 h p.i., the cell detachment of round cells led to the formation of large gaps throughout the monolayer, and enlarged vacuoles were observed in the infected cells. Furthermore, the dynamics of vacuolation induced by RGNNV were observed under phase microscopy. Small vacuoles could be observed in RGNNV-infected cells at 3 h p.i. The number of vacuoles was increased in the infected cells (from 3 h to 10 h p.i.), and small vacuoles were fused into large ones (from 6 h to 9 h p.i.) as the infection progressed (Figure 1B). Interestingly, the number of the dead cells stained by Image-It Dead green were significantly increased with the increase in infection time (Figure 1C). The cell mortality induced by RGNNV was approximately 15 and 52% at 24 h p.i. and 48 h p.i., respectively (Figure 1D).

**FIGURE 2:**
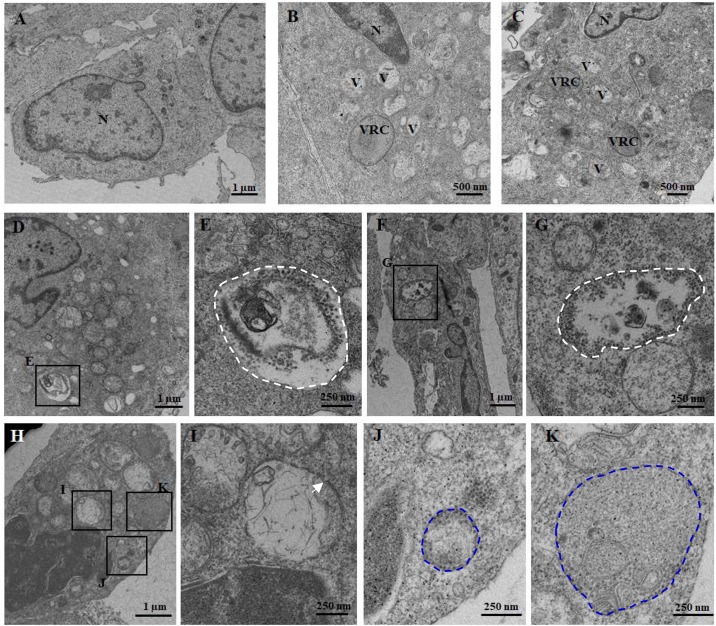
The ultrastructure of the vacuoles in mock **(A)** and RGNNV-infected cells **(B–K)**. **(A)** A normal nucleus, but not vacuoles, were observed in the mock-infected cells. **(B, C)** A condensed nucleus and numerous vacuoles were observed in the RGNNV-infected cells. “V” indicates vacuoles and “VRC” shows the virus replication center. **(D–G)** The white dotted circles show the vacuoles containing cell debris and viral particles. **(H–K)** The blue dotted circles show the monolayer membrane structures containing viruses, and the white arrows show the vacuoles with double-membrane structures.

To visualize the ultrastructure of cytoplasmic vacuoles, cells infected with RGNNV at 24 h were immediately fixed and observed using electron microscopy. As shown in Figure 2, an increased number of cytoplasmic vacuoles with various sizes were observed in RGNNV-infected GS cells. While few cytoplasmic vacuoles were observed in the mock-infected cells (Figures 2A–C). Interestingly, monolayer-membrane structures which contain numerous virus particles (viral replication compartments, VRCs) were observed in RGNNV-infected cells (Figures 2B,C). Viral particles approximately 30 nm in diameter were observed in some cytoplasmic vacuoles in addition to cell debris. The viral particles were primarily closed to the inner membrane of the vacuoles (Figures 2D–G). In addition, vacuoles with double- and single-membrane structures containing viruses were observed in the RGNNV-infected cells (Figures 2H–K).

### RGNNV Induced Cytoplasmic Vacuoles Derived From Lysosomes

**FIGURE 3:**
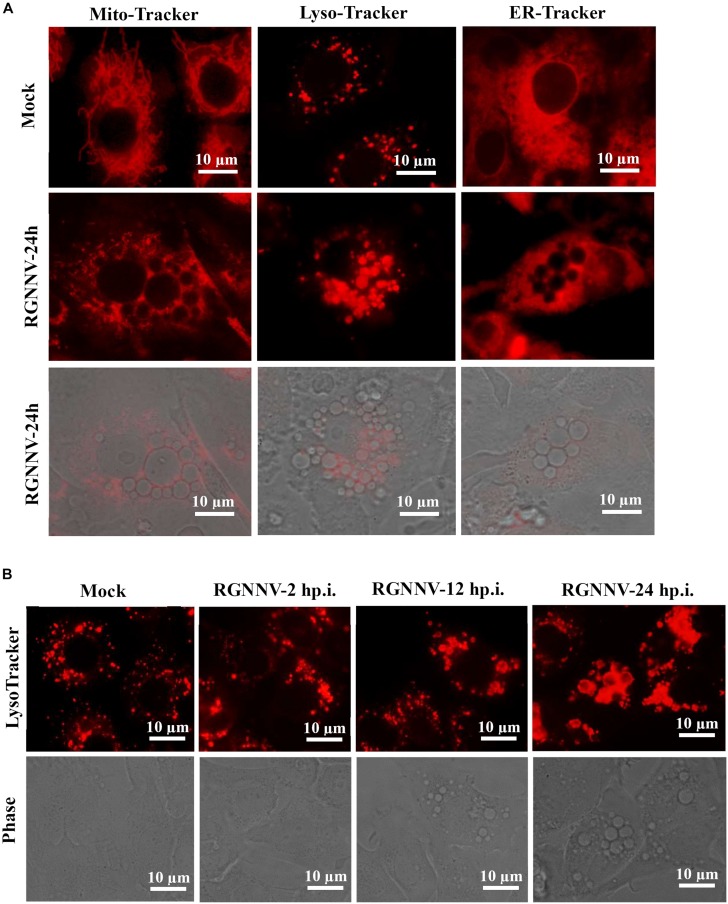
The RGNNV infection-induced cytoplasmic vacuoles were derived from endosome/lysosome organelles. **(A)** The status of the lysosome, endoplasmic reticulum (ER), and mitochondria in vacuolated cells. Mock- or RGNNV-infected cells were stained with Mito-Tracker, Lyso-Tracker, or ER-Tracker, respectively, and then observed under an immunofluorescence microscope. **(B)** The morphology of the lysosome and vacuoles at different time points during RGNNV infection.

To clarify the origin and composition of the vacuole, RGNNV-infected cells were stained with several organelle markers, including Mito-Tracker, Lyso-Tracker, and ER-Tracker. As shown in Figure 3A, the mitochondria in mock-infected cells exhibited a filamentous, elongated morphology, and the ER was evenly distributed throughout the cytoplasm. In contrast, the Lyso-Tracker-labeled vesicles were scattered throughout the cytoplasm. Following RGNNV infection, Mito-Tracker and ER-Tracker were excluded from the vacuoles and were not colocalized with the vacuoles, whereas Lyso-Tracker accumulated in the vacuoles. Thus, the results suggested that the vacuoles in the RGNNV-infected cells might be associated with the lysosomal compartments. To ascertain whether the endosome was associated with vacuolar membranes in RGNNV-infected cells, GS cells were transfected with pEGFP-Rab5 or pEGFP-Rab7, and subsequently infected with RGNNV. As shown in Supplementary Figure 1, the green fluorescence omitted from GFP-Rab5- or GFP-Rab7-transfected cells primarily resided on the vacuole membrane, which indicated that endosomal/lysosomal compartment organelles were involved in RGNNV infection-induced vacuole formation.

To detect the details of the lysosome dynamics during RGNNV infection, cells were infected with RGNNV at the indicated time points (2, 12, 24 h p.i.), stained with Lyso-Tracker, and observed under fluorescence microscopy. No obvious changes were observed in the lysosome morphology after RGNNV infection for 2 h compared to the control cells. From 12 to 24 h p.i., the fluorescence aggregates were enlarged and gathered in cytoplasmic vacuoles in RGNNV-infected cells. Accompanied by the severity of the vacuolation, the fluorescence aggregates were extremely enlarged and the majority had acuminated into the vacuoles (Figure 3B). This indicated that RGNNV infection significantly altered lysosome morphology, characterized by lysosome swelling. In combining the ultrastructure of the vacuoles during RGNNV infection, we speculate that the cytoplasmic vacuoles induced by RGNNV infection were derived from the lysosome.

### Cathepsin Activity Is Not Required for Vacuolization During RGNNV Infection

**FIGURE 4:**
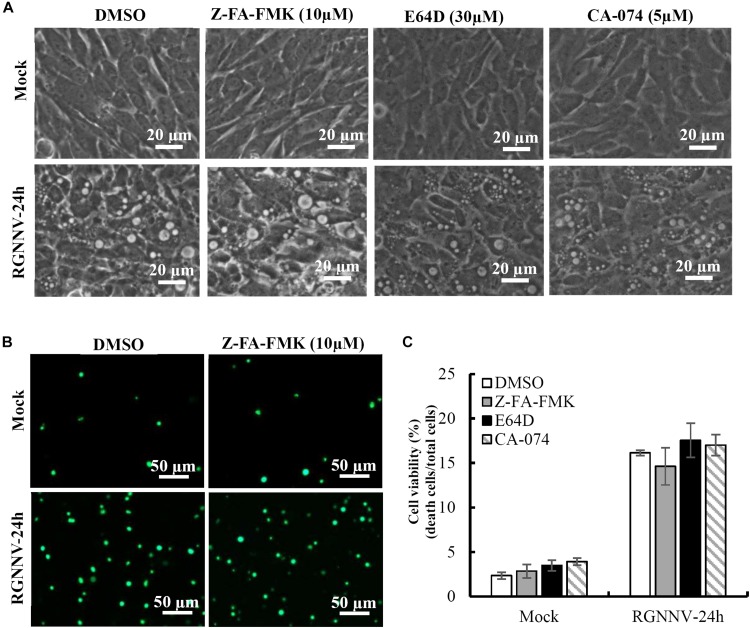
The effects of cathepsin inhibitors on vacuole formation during RGNNV infection. **(A)** The effects of different inhibitors on RGNNV infection-induced vacuolization. **(B)** The effect of Z-FA-FMK on cell death induced by RGNNV. **(C)** The percentage of cell death during RGNNV infection upon treatment with different inhibitors.

To clarify whether lysosomal cathepsins were involved in vacuolization and cell death induced by RGNNV infection, different cathepsin inhibitors were employed in this study, including Z-FA-FMK, CA-074, and E64D. As shown in Figure 4A, cells pretreated with Z-FA-FMK, CA-074, and E64D did not exhibit obvious effects on the formation of vacuoles induced by RGNNV infection. Consistent with this finding, RGNNV-induced cell death was not affected in the presence of Z-FA-FMK (Figure 4B). A quantitative analysis also showed that none of these inhibitors affected cell viability (Figure 4C). Thus, our results indicate that cathepsin activation was not essential for vacuole formation and the cell death induced by RGNNV infection.

### Lysosomal Acidification Is Required for RGNNV-Induced Vacuolization and Cell Death

**FIGURE 5:**
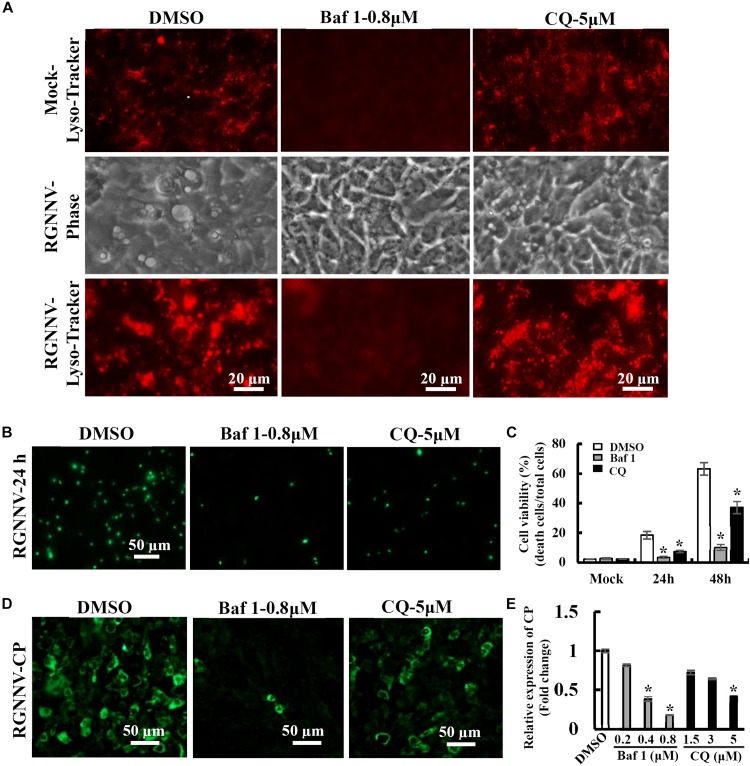
Vacuole formation and viral replication during RGNNV infection were dependent on lysosomal acidification. **(A)** The effects of bafilomycin A1 and CQ on lysosome morphology and vacuole formation. **(B)** The effect of bafilomycin A1 and CQ on RGNNV infection-induced cell death. **(C)** The percentage of cell death in RGNNV-infected cells following treatment with bafilomycin A1 or CQ. **(D)** Treatment with bafilomycin A1 or CQ significantly inhibited viral protein synthesis. **(E)** Quantitative analysis of RGNNV CP viral transcription.

To further verify whether the RGNNV-evoked vacuole formation was dependent on lysosomal acidification, bafilomycin A1 and chloroquine (CQ) were used to destroy lysosomal acidification. The effects of these inhibitors on vacuole formation were then subsequently determined. Bafilomycin A1, an inhibitor of vacuolar-type H^+^-ATPase, prevents the trafficking from early to late endosomes. Chloroquine is a lysosomotropic agent that prevents endosomal acidification. As expected, treatment with Bafilomycin A1 reduced Lyso-Tracker Red staining, which indicated that the lysosome structure was destroyed by Bafilomycin A1. Vacuolization induced by RGNNV was almost completely blocked by pretreatment with bafilomycin A1 compared with the DMSO-treated cells (Figure 5A). Interestingly, chloroquine treatment significantly inhibited vacuole fusion during RGNNV infection. Both bafilomycin A1 and chloroquine displayed a noticeable decrease in the cell death induced by RGNNV infection (Figures 5B,C). In addition, expression and transcription of the coat protein (CP) were also significantly inhibited in the presence of bafilomycin A1 or chloroquine in a dose-dependent manner (Figures 5D,E).

### Na^+^ and K^+^ Ionophore Exerts a Critical Role in Vacuole Formation

**FIGURE 6:**
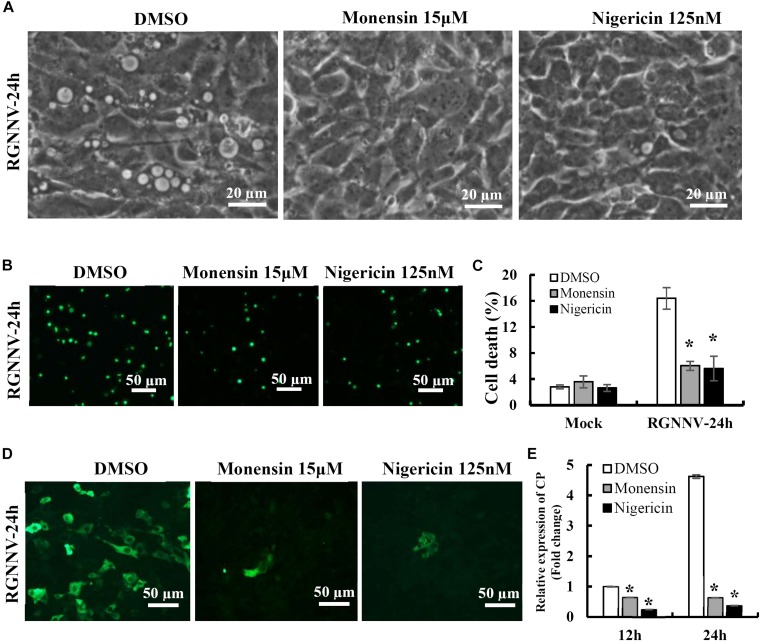
The Na+ and K+ ionophore exerted a vital role on vacuole formation. **(A)** The effects of nigericin and monensin treatment on vacuole formation during RGNNV infection. **(B)** The effect of nigericin and monensin on RGNNV infection-induced cell death. **(C)** The percentage of cell death in RGNNV-infected cells following treatment with nigericin and monensin. **(D)** Nigericin and monensin treatment significantly inhibited viral protein synthesis. **(E)** Quantitative analysis of the viral transcription of RGNNV CP.

To clarify the potential role of the proton gradient in the lysosomes during RGNNV infection, the effects of several ionophores on cytoplasmic vacuole formation and cell death were assessed. Nigericin and monensin, ionophores that uncouple the proton gradient present in lysosomes, were used in this study. As shown in Figure 6A, both monensin and nigericin were able to block the formation of cytoplasmic vacuoles induced by RGNNV. Moreover, treatment with monensin and nigericin significantly decreased the cell death induced by RGNNV compared to that of the DMSO-treated cells (Figures 6B,C). In addition, the transcription and protein synthesis of RGNNV CP both significantly weakened monensin or nigericin-treated infected cells compared to the DMSO-treated cells (Figures 6D,E). Thus, these data indicate that the Na^+^ and K^+^ imbalance play a vital role in the vacuolization and cell death evoked by an RGNNV infection.

**FIGURE 7:**
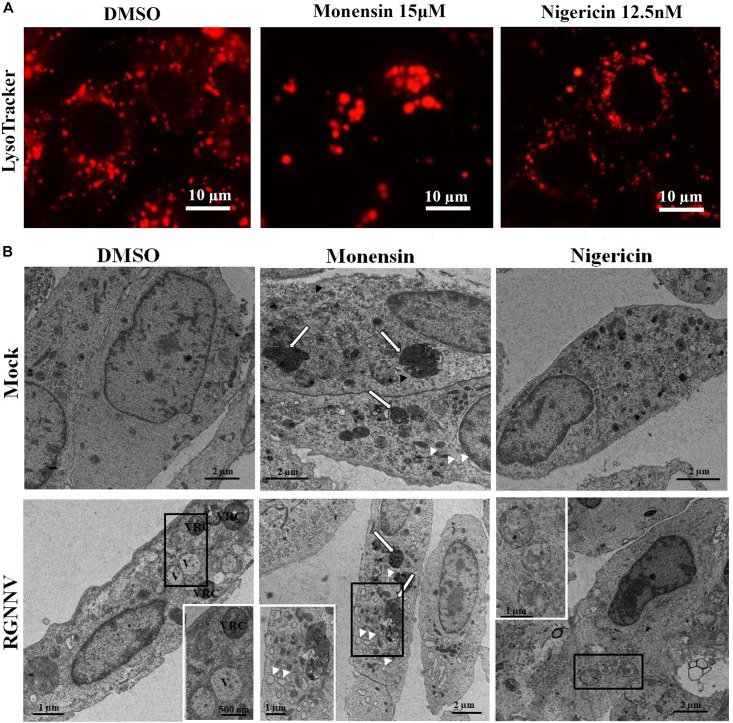
The effect of nigericin and monensin on the lysosome structure during RGNNV infection. **(A)** The lysosome structure upon nigericin and monensin treatment using fluorescence microscopy. **(B)** The lysosome ultrastructure in RGNNV-infected cells under electron microscopy.

To further verify whether monensin and nigericin affected the lysosome structure that subsequently blocked vacuole formation, the lysosome morphology in the monensin- and nigericin-treated cells were observed under fluorescence microscopy. Compared with DMSO treatment, monensin treatment resulted in an observed decrease in the number of lysosomal-labeled dots in the mock-infected cells, and the labeled lysosomes were clustered together in the cytoplasm. In contrast, nigericin treatment did not show an obvious effect on lysosome morphology compared to DMSO treatment (Figure 7A). In addition, the effects of monensin and nigericin treatment on the cellular ultrastructure was further assessed by electron microscopy. As shown in Figure 7B, dense granules surrounded by a monolayer, as well as small vacuoles about 100–150 nm in diameter, were observed in the monensin treated mock-infected cells. It is proposed that the dense granules induced by monensin treatment might be lysosome aggregates. Following RGNNV infection, cytoplasmic vacuoles were observed in the DMSO-treated infected cells, as well as VRCs, which contained numerous viral particles (Figure 7B). However, VRCs and scattered virions were not observed in the monensin- or nigericin-treated infected cells. This finding was consistent with the finding that both monensin and nigericin significantly inhibited viral CP expression. Although no obvious abnormal structures were present in the nigericin-treated cells, double-membraned organelles were observed in the cytoplasm in the RGNNV-infected nigericin-treated cells. However, whether these double membrane organelles were involved in affecting the formation of cytoplasmic vacuoles was uncertain.

### Autophagy Participates in RGNNV-Induced Vacuolization and Cell Death

**FIGURE 8:**
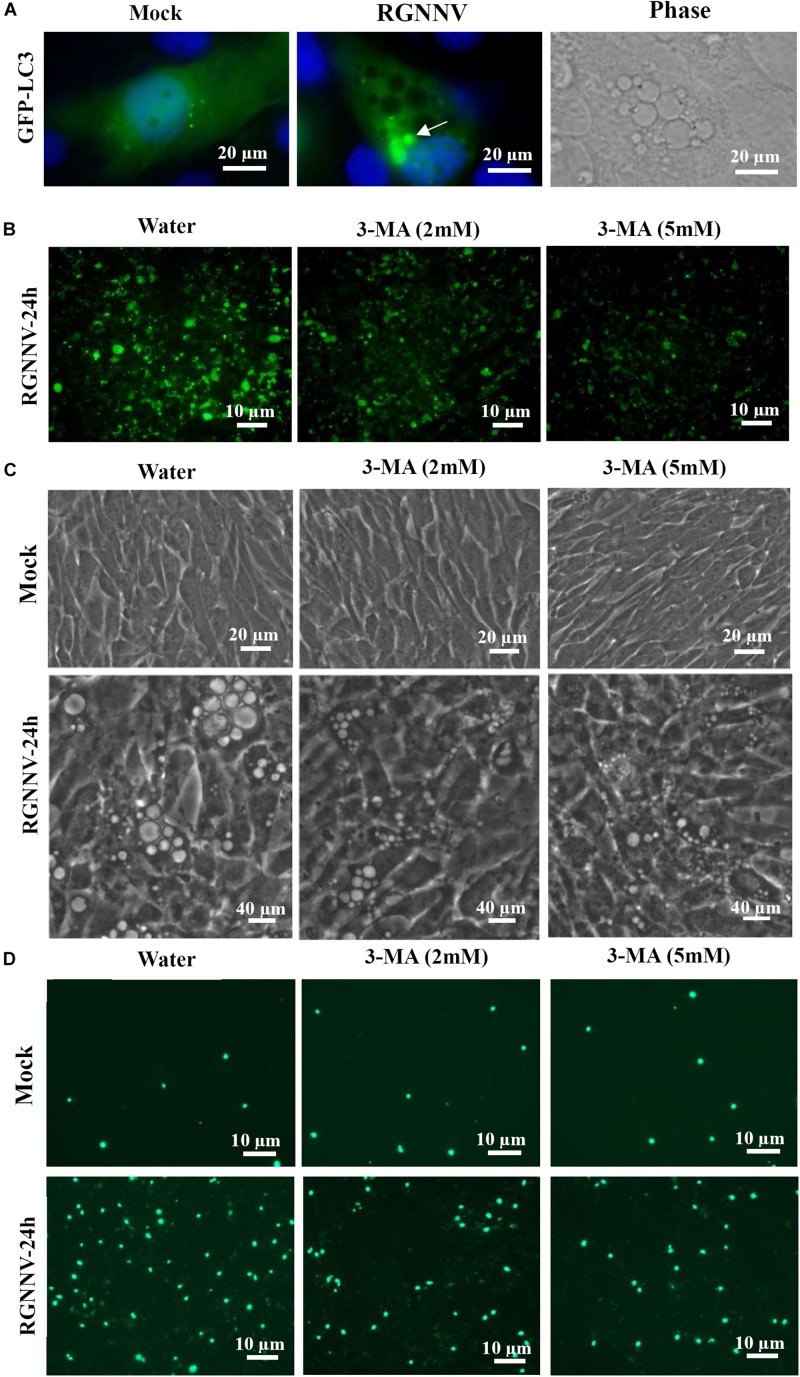
The role of autophagy in vacuole formation during RGNNV infection. **(A)** The localization patterns of LC3 during RGNNV infection-induced vacuolization. Arrow indicates the LC3 fluorescent puncta. **(B)** Detection of autophagy in RGNNV-infected cells under treatment with 3-MA. **(C)** The effects of 3-MA on RGNNV infection-induced vacuolization. **(D)** The effects of 3-MA on RGNNV infection-induced cell death.

To determine whether autophagy was involved in RGNNV-induced lysosomal vacuolation, we investigated the roles of autophagosomes during RGNNV-induced vacuolization. When autophagy is stimulated, cytosolic form of LC3 is conjugated to phosphatidylethanolamine to form LC3-phosphatidylethanolamine conjugate, which is recruited to autophagosomal membranes. In pEGFP-LC3 transfected cells, the punctate fluorescence signals (which primarily represent autophagosomes) can be observed under fluorescence microscope. As shown in Figure 8A, the pEGFP-LC3 fluorescence signals redistributed from a diffuse pattern in mock infected cells to a punctate cytoplasmic pattern in RGNNV-infected cells. Moreover, fluorescence spots were partially localized in the vacuolar lumen during RGNNV infection (Figure 8A). This suggested that autophagosomes might be involved in the vacuole formation induced by RGNNV. Next, 3-Methyladenine (3-MA), a drug which inhibits autophagy by blocking autophagosome formation via the inhibition of type III Phosphatidylinositol 3-kinases (PI-3K) was used in this study to further examine the role of autophagy during vacuole formation. As shown in Figure 8B, treatment with 3-MA decreased the number of fluorescently labeled cells during RGNNV infection. Of note, both the severity of the vacuolization and cell death induced by RGNNV were significantly weakened in the 3-MA-treated cells (Figures 8C,D). Collectively, these results suggest that autophagy participates in RGNNV-induced vacuolization and cell death.

## Discussion

As a major aquaculture pathogen of larval and juvenile marine finfish worldwide, NNV was found to induce the vacuolation and necrosis of the central nervous system (Ransangan and Manin, 2010; Doan et al., 2017; Yong et al., 2017). An *in vitro* NNV infection was found to evoke typical cytoplasmic vacuolization in a variety of cells, including grouper spleen (GS) and brain (GB) cells (Qin et al., 2006; Huang et al., 2011), striped snakehead fish cells (SSN-1) (Iwamoto et al., 2000), and European sea bass brain cells (DLB-1) (Chaves-Pozo et al., 2019). This indicates that cytoplasmic vacuolization caused by RGNNV was independent of the cell type. To our knowledge, the origin of the vacuoles evoked by RGNNV and the critical events during vacuolization remain poorly understood.

Increased evidence has found that numerous viruses can trigger cytoplasmic vacuolization (Shubin et al., 2016). Viral proteins (e.g., envelope or capsid proteins) typically act as inducers to trigger vacuole formation (Shubin et al., 2015; Luo et al., 2016). Although the origin of virus-induced vacuoles has not been fully characterized, several reports have demonstrated that the vacuoles evoked by different viruses may originate from different membrane organelles (e.g., ER and lysosomal organelles) (Shubin et al., 2016). In this report, double-membrane structures in the cytoplasmic vacuoles induced by RGNNV were observed under electron microscopy. The temporal analysis indicated that small cytoplasmic vacuoles were present during the early stages of RGNNV infection, some of which fused into one large cell as the infection progressed. Further analysis showed that both Mito-Tracker and ER-Tracker were excluded from vacuoles, whereas Lyso-Tracker had accumulated in the vacuoles of the RGNNV-infected cells, and the vacuolar membranes simultaneously labeled the endosome markers, Rab5 and Rab7. Moreover, the increase in lysosome volume was observed to be accompanied by the occurrence of cytoplasmic vacuoles. Thus, we speculated that RGNNV-induced vacuoles might originate from the endosomal/lysosomal compartments, rather than the mitochondria and ER. Bovine viral diarrhea virus and SV40 also induced the vacuolization of acidic endosomal-lysosomal organelles in infected cells (Birk et al., 2008; Luo et al., 2016). In addition, vacuolization of different intracellular compartments always indicates the pathological status and accompanies different types of cell death (Shubin et al., 2015; Monel et al., 2017). Our results also show that the proportion of cell death at different time points was consistent with the severity of vacuolization, suggesting that RGNNV-triggered vacuolization of lysosomal/endosomal organelles was accompanied by cell death during viral infection.

As an important cellular organelle, lysosomes maintain an acidic luminal pH for the purpose of degrading internalized macromolecules and lysosomal proteases (e.g., cathepsins exert a crucial role in maintaining cell metabolism homeostasis and participate in different types of cell death (Repnik et al., 2012; Mauvezin et al., 2015). The V-ATPase inhibitor bafilomycin A was able to completely block vacuolization in RGNNV-infected cells, indicating that RGNNV-induced vacuoles also required V-ATPase activity. Moreover, chloroquine, a lysosomotropic agent that prevents endosomal acidification, also significantly inhibited the fusion of the vacuole formation during RGNNV infection. Thus, we speculated that the maintenance of lysosome acidification is required for RGNNV-induced vacuolization and cell death. To clarify the role of cathepsins in RGNNV-induced vacuolization, different cathepsin inhibitors, including Z-FA-FMK, CA-074, and E64D, were used in this study. Interestingly, none of these selected inhibitors showed obvious effects on RGNNV-triggered vacuolization and cell death. This suggests that multiple cathepsins (i.e., cathepsins B, L, and K) were not involved in this process.

Cytoplasmic vacuolization always accompanies different types of cell death, including autophagy, paraptosis-like cell death, and necroptosis (Kar et al., 2009; Huang et al., 2011; Singha et al., 2013). When autophagy occurs, LC3 becomes conjugated to phosphatidylethanolamine at autophagosome-forming sites, and redistributes from a diffuse pattern to a punctate cytoplasmic pattern (Kabeya et al., 2000; Tanida et al., 2008). Given that our previous studies indicated that RGNNV infection could induce autophagy (Huang et al., 2015), we raised the question of whether autophagy was involved in lysosomal vacuolization during RGNNV infection. To address this issue, we first examined whether autophagosome-like vesicles and the altered distribution for LC3 proteins occurred in vacuolization. The results showed that the autophagic vacuoles containing intact cytoplasmic material and viral particles, and the small amount of LC3 proteins, were observed in the lumen of RGNNV-induced cytoplasmic vacuoles, suggesting that autophagosomes were involved in RGNNV infection. Secondly, we found that 3-MA treatment had remarkable inhibitory effects on RGNNV-induced vacuolation and cell death. In addition, the Bafilomycin A1-mediated inhibition on the number and volume of vacuoles might provide further evidence of the involvement of autophagy in vacuolization. This is because Bafilomycin A1 can also function as an inhibitor of late phase of autophagy by preventing the fusion between autophagosomes and lysosomes, as well as lysosomal degradation (Kissing et al., 2015; Mauvezin et al., 2015). Based on these findings, we speculate that autophagy participates in RGNNV-induced lysosomal vacuolization.

**FIGURE 9:**
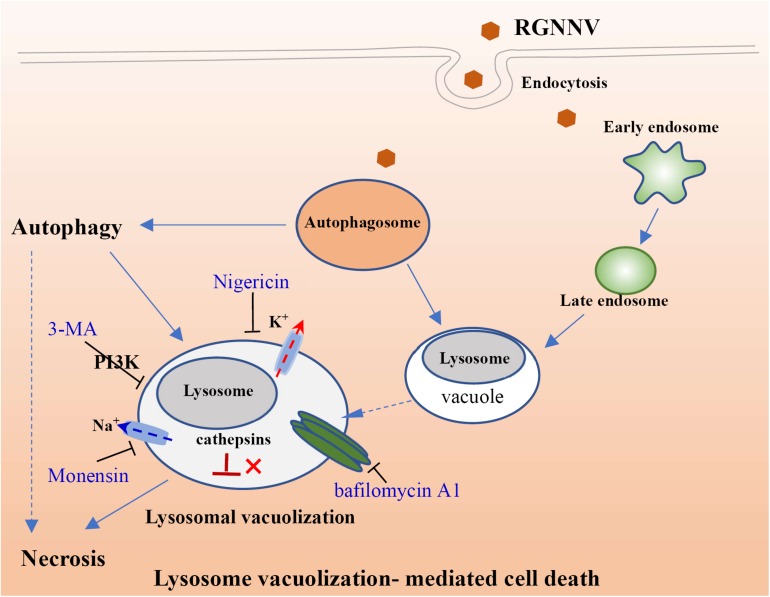
Proposed model of lysosomal vacuolization-mediated cell death during RGNNV infection.

In addition to V-ATPase activity, the proton-selective ionophore, monensin (Na^+^/H^+^), as well as nigericin (K^+^/H^+^), have been demonstrated to raise the pH of the acidified compartments, and inhibit autophagosome-lysosome fusion (Lim et al., 2012; Yoon et al., 2013). In this study, we elucidated an important role of intracellular ionophores in the lysosomal vacuolization induced by RGNNV infection. Our data show that treatment with both monensin and nigericin have observable effects on cytoplasmic vacuolization in RGNNV-infected cells. Combined with the inhibitory effect of Bafilomycin A1, our results further demonstrated that the maintenance of lysosome acidic was required for RGNNV infection-induced vacuolization and cell death (Figure 9). Moreover, alterations in the lysosome structure (e.g., lysosome swelling) were also observed in monensin-treated cells. Thus, we presumed that in the process of RGNNV infection, acidic lysosomes were first fused with late endosomes to form cytoplasmic vacuoles, which was followed by lysosome swelling. The results from electron microscopy also showed that viral particles were present in some cytoplasmic vacuoles in conjunction with cell debris. Given the critical role of lysosomes in acidic degradation, we speculated that the cytoplasmic vacuoles derived from endosomal/lysosomal organelles might be an important host defense strategy for the degradation of viral proteins or virion clearance.

In conclusion, the findings of this study suggest that RGNNV-induced vacuoles originate from endosomal/lysosomal organelles and evoke an alteration of the lysosomal structure. Moreover, V-ATPase activity and the balance of the intracellular ionophore, but not cathepsin activation are essential for RGNNV infection-induced lysosomal vacuolization and cell death. In addition, autophagy might exert a critical role on vacuole formation and cell death during RGNNV infection (Figure 9). Taken together, we propose that autophagy participates in RGNNV infection-induced lysosomal vacuolization and cell death. Thus, our data will provide novel insight into our understanding of the molecular mechanisms of nodavirus pathogenesis.

## Data Availability Statement

All datasets generated for this study are included in the article/Supplementary Material.

## Author Contributions

XH and YH carried out the main experiments, analyzed the data, and drafted the manuscript. YZ and ZL participated in the qPCR experiments and trypan blue staining. JZ participated in the immunofluorescence experiment. CL prepared the ultrathin sections. XH and QQ designed the experiments and reviewed the manuscript. All authors read and approved the final manuscript.

## Conflict of Interest

The authors declare that the research was conducted in the absence of any commercial or financial relationships that could be construed as a potential conflict of interest.

## Supplementary Material

FIGURE S1 Rab5 and Rab7 localization during RGNNV infection. GS cells were transfected with pEGFP-Rab5 or pEGFP-Rab7, and subsequently infected with RGNNV. At 24 h p.i., the cells were stained with DAPI and the fluorescence was observed via fluorescence microscopy.
