# Supplementary figures and images for: Autophagy Participates in Lysosomal Vacuolation-Mediated Cell Death in RGNNV-Infected Cells
Source: Front Microbiol. 2020 Apr 30;11:790. doi: 10.3389/fmicb.2020.00790 (PMC7212415; doi:10.3389/fmicb.2020.00790)

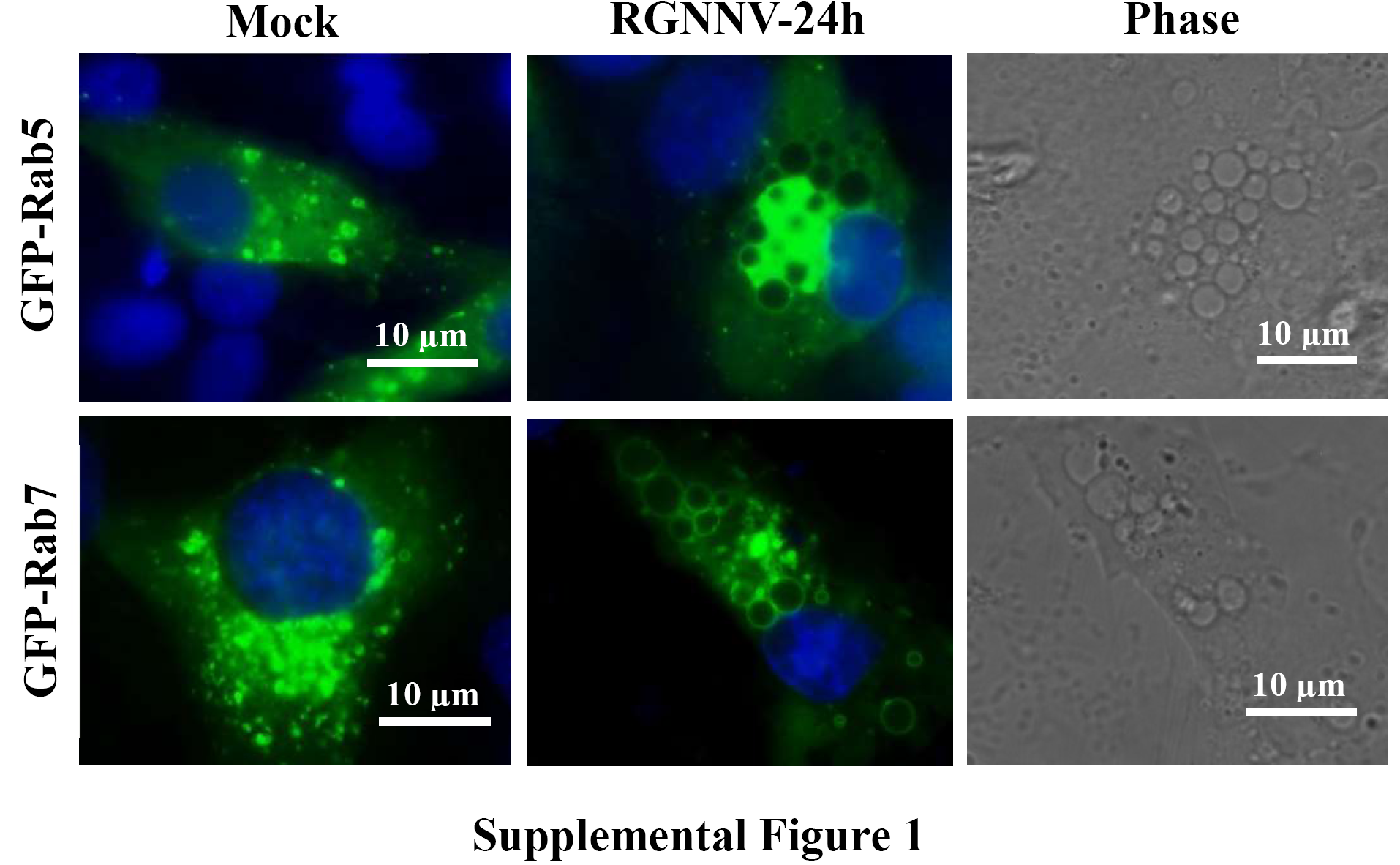

Supplement: FIGURE S1 — Rab5 and Rab7 localization during RGNNV infection. GS cells were transfected with pEGFP-Rab5 or pEGFP-Rab7, and subsequently infected with RGNNV. At 24 h p.i., the cells were stained with DAPI and the fluorescence was observed via fluorescence microscopy. [file Image_1.TIF]
